# Membralin Assembles a MAN1B1–VCP Complex to Target Foreign Glycoproteins from the Endoplasmic Reticulum to Lysosomes for Degradation

**DOI:** 10.1002/advs.202519256

**Published:** 2025-12-01

**Authors:** Jing Zhang, Xiaoran Lu, Sunan Li, Tao Wang, Iqbal Ahmad, Yong‐Hui Zheng

**Affiliations:** ^1^ State Key Laboratory for Animal Disease Control and Prevention Harbin Veterinary Research Institute Chinese Academy of Agricultural Sciences Harbin 150069 China; ^2^ Department of Microbiology and Immunology The University of Illinois Chicago Chicago IL 60612 USA

**Keywords:** ER‐phagy receptor, MAN1B1, membralin, reticulophagy, TMEM259, viral glycoprotein

## Abstract

Protein quality control in the endoplasmic reticulum (ER) maintains proteostasis by eliminating aberrant or foreign proteins through ER‐associated degradation (ERAD) or ER‐to‐lysosome‐associated degradation (ERLAD). Here, Membralin (TMEM259) is identified as a previously unrecognized ER‐phagy receptor that assembles a selective degradation machinery targeting viral class I fusion glycoproteins. Membralin recruits MAN1B1, an α‐mannosidase that trims high‐mannose N‐glycans, through its luminal loop, and VCP/p97 through its cytoplasmic loop, while its cytoplasmic tail contains a functional LC3‐interacting region (LIR) essential for autophagic delivery. This Membralin–MAN1B1–VCP axis directs viral glycoproteins such as SARS‐CoV‐2 spike, Ebola GP, influenza HA, and HIV‐1 Env to lysosomes for degradation independently of polyubiquitination or canonical ER‐phagy receptors. In contrast, misfolded host glycoproteins are degraded through conventional ERAD or FAM134B‐dependent ERLAD pathways. Mechanistically, the Membralin complex selectively recognizes densely glycosylated substrates, likely by sensing clustered N‐glycans characteristic of viral envelope proteins. Loss of Membralin or MAN1B1 markedly enhances pseudoviral infectivity, underscoring its antiviral role. These findings reveal a ubiquitin‐independent ERLAD pathway that discriminates foreign from host glycoproteins and establish Membralin as a central scaffold coordinating ER quality control and innate antiviral defense.

## Graphical abstract

This study identifies Membralin as an ER‐phagy receptor that recruits MAN1B1 and VCP to form a selective ERLAD complex. By sensing dense N‐glycan clusters on viral fusion glycoproteins, this ubiquitin‐independent pathway directs SARS‐CoV‐2 spike, Ebola GP, influenza HA, and HIV‐1 Env to lysosomal degradation, thereby limiting viral infectivity.

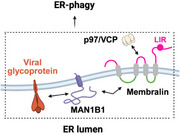

## 1 Introduction

The endoplasmic reticulum (ER) is essential for protein synthesis, producing both secreted and integral membrane proteins. It plays a critical role in ensuring that newly synthesized proteins are properly folded into their native conformations. When terminally misfolded proteins accumulate, the ER experiences stress, which can be mitigated by the ER quality control (ERQC) systems, including ER‐associated degradation (ERAD) and ER‐to‐lysosome‐associated degradation (ERLAD).^[^ ^1^ ^]^

Mannosyl‐oligosaccharide 1,2‐α‐mannosidase (MAN1B1) is a key enzyme in the ERAD process that specifically targets glycoproteins by co‐opting with three ER degradation‐enhancing α‐mannosidase‐like (EDEM) proteins.^[^ ^2^ ^]^ During glycoprotein folding in the ER, MAN1B1 or EDEM2 removes a single 1,2‐α‐linked mannose residue from Man9GlcNAc2 on N‐glycan precursors, yielding Man8GlcNAc2.^[^ ^3^, ^4^, ^5^ ^]^ For terminally misfolded glycoproteins, MAN1B1, EDEM1, or EDEM3 further trims two or three additional 1,2‐α‐linked mannose residues, producing Man(5–6)GlcNAc2.^[^ ^4^, ^6^, ^7^ ^]^ Glycoproteins with these low‐mannose N‐glycans are recruited by lectins osteosarcoma amplified 9 (OS9) and XTP3‐transactivated gene B protein (XTP3‐B), retrotranslocated, and degraded in proteasomes via the ERAD pathway.^[^ ^8^ ^]^

ERLAD directly segregates misfolded proteins in ER subdomains that detach from the ER and targets ERAD‐resistant substrates to endolysosomes for degradation.^[^ ^9^ ^]^ This process may involve ER‐phagy receptors on the ER membrane, including reticulophagy regulator 1 (RETREG1, or FAM134B), RETREG1‐2, RETREG2 (FAM134A), RETREG3 (FAM134C), TEX264, RTN3L, ATL3, CCPG1, and SEC62. RETRER1‐2 is an N‐terminally truncated isoform of RETRER1. They are embedded in the ER membrane and are characterized by at least one cytosolic LC3‐interacting region (LIR). FAM134B and RTN3L also contain a reticulon‐homology domain (RHD) within the ER membrane.

Membralin/TMEM259, encoded by *TMEM259*, is crucial for motor neuron survival as it maintains optimal expression levels of nicastrin, a core component of the γ‐secretase complex.^[^ ^10^ ^]^ It is a multi‐pass membrane protein in the ER and implicated in alleviating ER stress by interacting with proteins in the ERAD network.^[^ ^11^ ^]^

Class I fusion proteins, such as human immunodeficiency virus type 1 (HIV‐1) envelope glycoprotein (Env), influenza A virus (IAV) hemagglutinin (HA), Ebola virus (EBOV) glycoprotein (GP), and severe acute respiratory syndrome coronavirus 2 (SARS‐CoV‐2) spike (S) protein, play crucial roles in the entry of these viruses.^[^ ^8^ ^]^ Although these proteins do not share sequence homology, they exhibit a common trimeric‐hairpin structure with a central coiled coil covered by a dense glycan shield. Class I fusion proteins are expressed as type I transmembrane polypeptide precursors, folded in the ER, and cleaved by furin in the Golgi. We have previously shown that the expression of HIV‐1‐Env, EBOV‐GP, and IAV‐HA induces ER stress and undergoes MAN1B1‐mediated degradation.^[^ ^12^, ^13^, ^14^, ^15^, ^16^, ^17^ ^]^

Here, we demonstrate that Membralin orchestrates a specialized protein‐degradation complex on the ER membrane to initiate ERLAD. Acting as a scaffold, Membralin recruits MAN1B1 through its luminal loop and p97/VCP through its cytoplasmic loop, and its cytoplasmic tail harbors a functional LIR motif. This ER‐resident machinery selectively targets class I viral fusion proteins, but not conventional misfolded host proteins, likely by recognizing densely glycosylated N‐glycan structures.

## 2 Results

### 2.1 Human Coronavirus Spike Proteins are MAN1B1 Substrates

SARS‐CoV‐2 (SARS2) infection induces ER stress, significantly contributing to COVID‐19 cytopathic and inflammatory pathogenesis.^[^ ^18^, ^19^, ^20^ ^]^ During SARS‐CoV (SARS1) infection, its spike protein plays a significant role in the induction of ER stress compared to other viral proteins.^[^ ^21^ ^]^ Thus, we investigated how SARS2‐S activates UPR and triggers its relevant signaling events, including SARS1 and Middle East respiratory syndrome coronavirus (MERS) spike proteins as controls.

During ER stress, binding‐immunoglobulin protein (BiP) is upregulated to serve as a master ER stress regulator, resulting in the activation of three ER transmembrane receptors: the protein kinase R (PKR)‐like endoplasmic reticulum kinase (PERK), inositol‐requiring enzyme type 1 (IRE1), and activating transcription factor 6 (ATF6). PERK activates a transcription factor ATF4, and IRE1 activates another transcription factor, X‐box binding protein 1 (XBP1). As we reported, we used luciferase based reporter assays to detect the upregulation and activation of BiP, XBP1, ATF4, and ATF6.^[^ ^12^, ^14^ ^]^ As a positive control for ER stress, the terminally misfolded human Serpin A1 (SERPINA1)/alpha1‐antitrypsin (AAT) variant, null (Hong Kong) (NHK), was used.^[^ ^22^ ^]^

**Figure 1:**
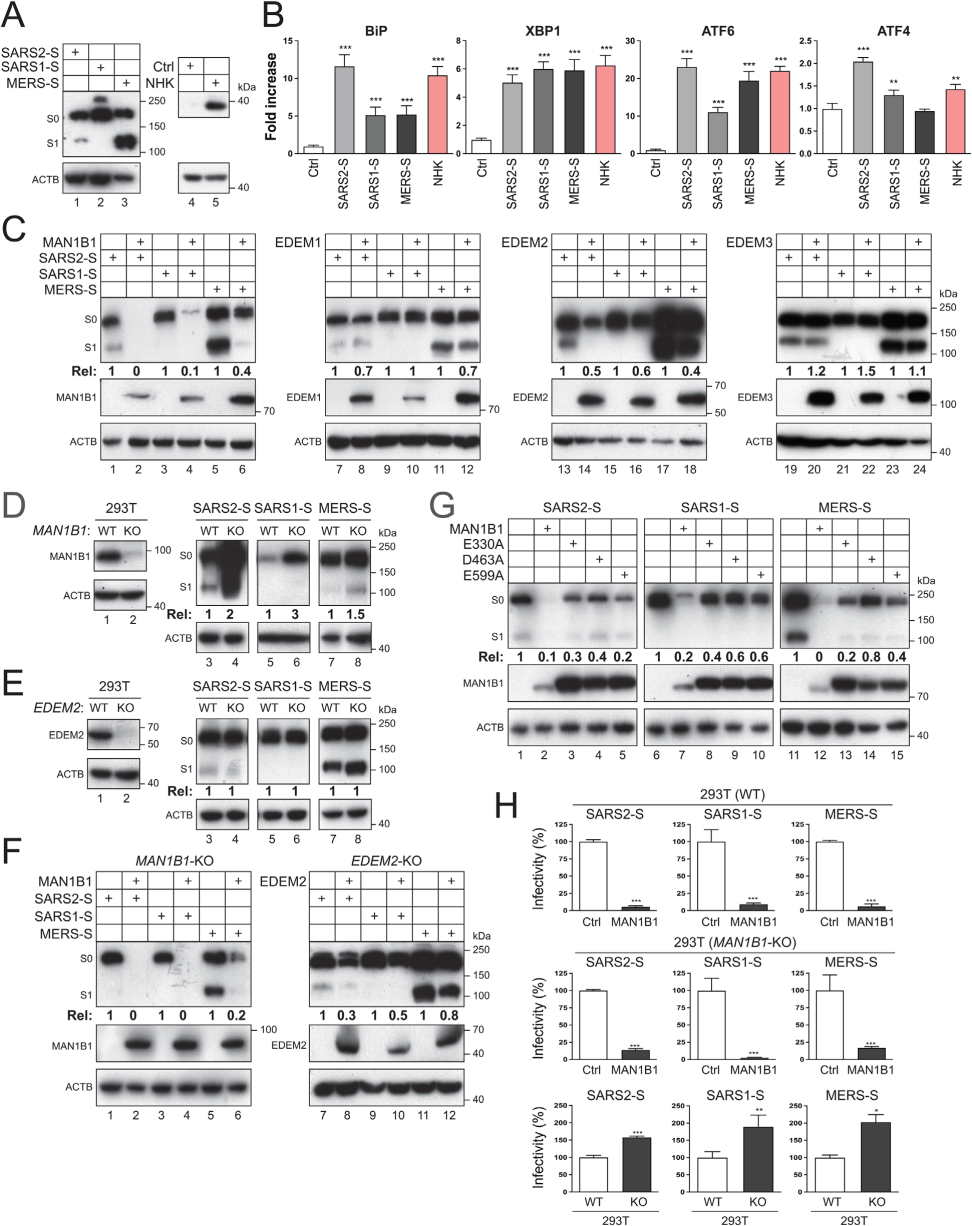
Human coronavirus spike proteins are MAN1B1 substrates. A) SARS1‐S, SARS2‐S, and MERS‐S proteins with a FLAG‐tag, and NHK were expressed in HEK293T cells and detected by WB. The unprocessed S_0_ precursor and processed S_1_ subunit are indicated. β‐actin (ACTB) was used as a loading control. The viral proteins were detected by anti‐FLAG, and NHK was detected by anti‐AAT antibodies. B) Spike and NHK proteins were expressed with ER‐stress luciferase‐reporter vector pLightSwitch‐BiP, p5×ATF6‐GL3, pXBP1u‐FLuc, or pATF4‐UTR‐Fluc in HEK293T cells. Luciferase activity was measured and are presented as a relative value, with the activity in the presence of a control vector (Ctrl) set as 1. C) Spike proteins were expressed with MAN1B1, EDEM1, EDEM2, or EDEM3 with an HA‐tag in HEK293T cells, and their expression was detected by WB. D) Spike proteins were expressed in HEK293T wild‐type (WT) and *MAN1B1*‐knockout (KO) cells, and their expression was compared by WB. E) Spike proteins were expressed in HEK293T WT and *EDEM2*‐KO cells, and their expression was compared by WB. F) Spike proteins were expressed with MAN1B1 and EDEM2 in HEK293T *MAN1B1*‐KO or *EDEM2*‐KO cells, and their expression was compared by WB. G) Spike proteins were expressed with MAN1B1 and its catalytic site‐deficient mutants (E330A, D463A, E599A) in *MAN1B1*‐KO cells, and their expression was analyzed by WB. H) HIV‐1 FLuc‐reporter pseudovirions (PVs) expressing coronavirus spike proteins were produced in the presence and absence of ectopic MAN1B1 from HEK293T WT or *MAN1B1*‐KO cells. After infecting Huh7 cells with an equal number of PVs, viral infection was determined by measuring intracellular luciferase activities. Results are presented as relative values, with the activities in the presence of a control vector set as 100. The levels of spike protein expression in (C), (D), (E), (F), and (G) were quantified by Image J and expressed as relative (Rel) values. Error bars in (B) and (H) represent the standard error of measurements (SEMs) calculated from three experiments (*n* = 3). *p < 0.05, **p < 0.01, ***p < 0.001, ****p < 0.0001, ns (not significant, *p* > 0.05). All experiments were repeated three times, and representative results are shown.

SARS1‐S, SARS2‐S, MERS‐S, and NHK were expressed with these different reporters, and levels of ER stress were determined. SARS1‐S, SARS2‐S, MERS‐S, and NHK expression was detected and confirmed by western blotting (WB) (**Figure** **1**A). As expected, the spike protein precursor S_0_ and its furin‐processed S_1_ subunit were detected from SARS2 and MERS, but not SARS1, because SARS1‐S does not contain a furin cleavage site. In addition, NHK upregulated BiP and activated ATF6, XBP1, and ATF4 (Figure 1B). Like NHK, SARS2‐S also did so at comparable levels. SARS1‐S and MERS‐S strongly activated XBP1, but their upregulation and activation of BiP and ATF4 were lower than SARS2‐S and NHK. Unlike SARS1‐S, MERS‐S also significantly activated ATF6. Thus, all these spike proteins activate ER stress, but the SARS2‐S activity is much broader and more potent.

Previously, we identified HIV‐1‐Env, IAV‐HA, and EBOV‐GP as MAN1B1 substrates for degradation.^[^ ^12^, ^14^, ^16^ ^]^ When SARS1‐S, SARS2‐S, or MERS‐S were expressed with MAN1B1, EDEM1, EDEM2, and EDEM3, MAN1B1 lowered all these viral protein expressions at steady‐state, whereas EDEM1, EDEM2, and EDEM3 had little effect (Figure 1C). We then knocked out *MAN1B1* and *EDEM2*. When SARS1‐S, SARS2‐S, and MERS‐S were expressed in wild‐type (WT) and these knockout (KO) cells, their expression was increased in *MAN1B1*‐KO cells (Figure 1D), but not *EDEM2*‐KO cells (Figure 1E). Next, ectopic MAN1B1 and EDEM2 were expressed with the individual spike proteins in these KO cells. The MAN1B1 inhibitory activity of spike protein expression was restored, but EDEM2 had little activity (Figure 1F).

MAN1B1 has three critical catalytic sites (E330, D463, and E599), which are conserved among class I α‐mannosidases. When these residues were mutated to alanine, the MAN1B1 inhibitory activity was disrupted, indicating that its enzymatic activity is required (Figure 1G).

To demonstrate that MAN1B1 has antiviral activity, HIV‐1 firefly luciferase (FLuc)‐reporter pseudovirions (PVs) expressing SARS1‐S, SARS2‐S, and MERS‐S were produced from HEK293T WT and *MAN1B1*‐KO cells in the presence of ectopic MAN1B1, and their infectivity was determined by infection of Huh7 cells. MAN1B1 strongly inhibited the infectivity of all three PVs up to 40‐fold (Figure 1H). We also compared the infectivity of PVs produced from these cells in the absence of ectopic MAN1B1. PVs from the KO cells showed higher infectivity than those from WT cells (Figure 1H), confirming that the endogenous MAN1B1 has antiviral activity.

### 2.2 Class I Fusion Proteins are Degraded via ERLAD Independent of Polyubiquitination

**Figure 2:**
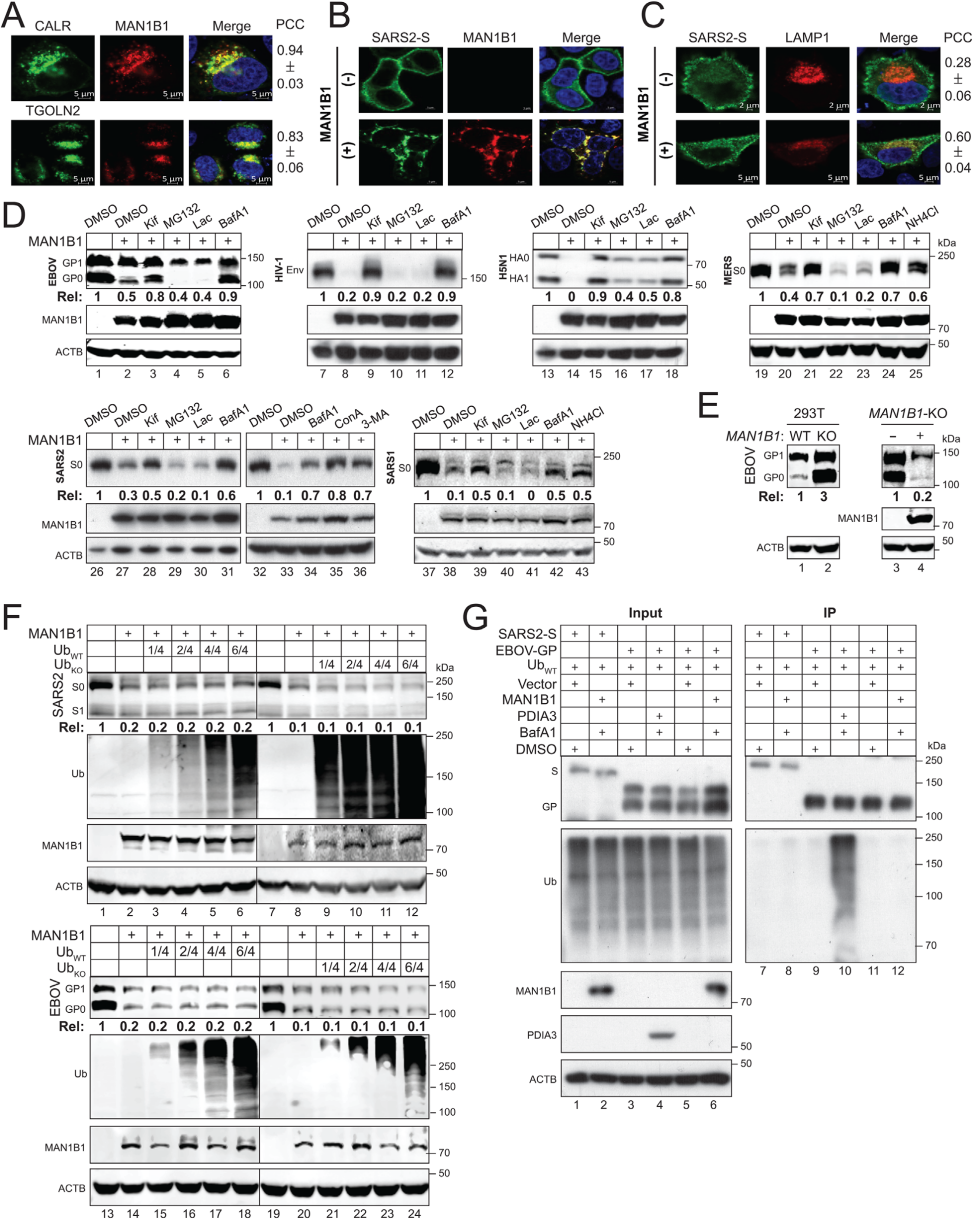
MAN1B1 targets class I fusion proteins for lysosomal degradation independent of polyubiquitination. A) MAN1B1‐mCherry‐tag and CALR or TGOLN2 with a GFP‐tag were expressed in HeLa cells, and their colocalization was investigated by confocal microscopy (scale bar 5 µm). PCC, Pearson's correlation coefficient. B) SARS2‐S‐GFP was expressed with MAN1B1‐mCherry in HeLa cells, and their subcellular localization was determined by confocal microscopy (scale bar 5 µm). C) SARS2‐S‐GFP was expressed with LAMP1‐mCherry in the presence or absence of MAN1B1 in HeLa cells. The co‐localization of SARS2‐S with LAMP1 was determined by confocal microscopy (scale bar 2 or 5 µm). D) EBOV‐GP, HIV‐1‐Env, H5N1‐HA, SARS2‐S, SARS1‐S, and MERS‐S were expressed with MAN1B1 in HEK293T cells and treated with 50 µm Kif, 20 µm MG132, 20 µm Lac, 100 nm BafA1, 20 mm NH_4_Cl, 20 nm ConA, or 10 mm 3‐MA. Protein expression was determined by WB. E) EBOV‐GP was expressed in HEK293T WT and *MAN1B1*‐KO cells, or EBOV‐GP was expressed in HEK293T *MAN1B1*‐KO cells in the presence or absence of ectopic MAN1B1 expression. EBOV‐GP expression was compared by WB. F) SARS2‐S and EBOV‐GP were expressed with MAN1B1 in HEK293T cells in the presence of increasing amounts of Ub_WT_ or Ub_KO_. Protein expression was determined by WB. G) SARS2‐S and EBOV‐GP with a FLAG‐tag were expressed with Ub_WT_ and MAN1B1 or PDIA3 in HEK293T cells. Cells were treated with DMSO or 100 nm BafA1. Proteins were immunoprecipitated (IP) with anti‐FLAG and analyzed by WB. The levels of class I fusion protein expression in (D), (E), and (F) were quantified by Image J and expressed as Rel values. All experiments were repeated three times, and representative results are shown.

We investigated how MAN1B1 affects the subcellular localization of SARS2‐S by confocal microscopy. MAN1B1 with a mCherry‐tag was expressed with the ER marker calreticulin (CALR), or the Golgi marker *trans*‐Golgi network integral membrane protein 2 (TGOLN2) with a GFP‐tag. MAN1B1 colocalized with both CALR and TGOLN2, with similar values of the Pearson's correlation coefficient (PCC) (**Figure** **2**A), reflecting a dynamic MAN1B1 cycling between these two organelles as reported.^[^ ^23^, ^24^ ^]^ When SARS2‐S with a GFP‐tag was expressed alone, it was predominantly found on the plasma membrane, and some SRAS2‐S proteins were also found in the cytoplasm. However, when it was expressed with MAN1B1‐mCherry, it was predominantly found in intracellular compartments and colocalized with MAN1B1 (Figure 2B). SARS2‐S‐GFP was then expressed with the lysosome marker, lysosomal‐associated membrane protein 1 (LAMP1) with a mCherry‐tag. SARS2‐S colocalized with LAMP1 inside cells only when MAN1B1 was also expressed (Figure 2C), indicating that SARS2‐S is recruited to lysosomes.

To demonstrate protein degradation, class I fusion proteins, including EBOV‐GP, HIV‐1‐Env, IAV (H5N1)‐HA, SARS2‐S, SARS1‐S, and MERS‐S were expressed with MAN1B1 in HEK293T cells and treated with different types of inhibitors, including class I α‐mannosidase inhibitor kifunensine (Kif), proteasomal inhibitors MG132 and lactacystin (Lac), lysosomal inhibitors NH_4_Cl and bafilomycin A1 (BafA1), and autophagy inhibitors concanamycin A (ConA) and 3‐methyladenine (3‐MA). As expected, Kif blocked the MAN1B1 activity to inhibit class I fusion protein expression (Figure 2D, lanes 3, 9, 15, 21, 28, 39), and neither MG132 nor Lac did so, indicating that they are degraded in the absence of proteasomes. However, NH_4_Cl, BafA1, ConA, and 3‐MA all blocked the MAN1B1 inhibitory activity, indicating that they are degraded by autophagy/lysosomes. To further confirm the endogenous MAN1B1 activity, EBOV‐GP was expressed again in WT and *MAN1B1*‐KO cells. EBOV‐GP expression was greatly increased in KO cells, which was countered by ectopic MAN1B1 (Figure 2E).

Next, we determined whether polyubiquitination is involved in this degradation process. We reported that ER‐chaperones such as disulfide‐isomerase A3 (PDIA3) target EBOV‐GP for ER‐phagy in a polyubiquitination‐dependent manner.^[^ ^14^, ^25^ ^]^ When SARS2‐S and EBOV‐GP were expressed with MAN1B1 and increasing amounts of WT ubiquitin (Ub_WT_) or its lysine‐free mutant (Ub_KO_), neither influenced their downregulation (Figure 2F).

SARS2‐S and EBOV‐GP were then expressed with MAN1B1 in the presence of Ub_WT_, and SARS2‐S and EBOV‐GP polyubiquitination were detected by immunoprecipitated (IP), including PDIA3 as a control. To prevent SARS2‐S and EBOV‐GP degradation triggered by MAN1B1 and PDIA3, cells were treated with BafA1 (Figure 2G, lanes 2, 4, 6). Only PDIA3 promoted EBOV‐GP polyubiquitination, whereas MAN1B1 did not (lanes 9, 10, 11, 12). Additionally, MAN1B1 did not induce SARS2‐S polyubiquitination either (lanes 7, 8). Thus, MAN1B1 does not trigger SARS2‐S and EBOV‐GP polyubiquitination.

### 2.3 Membralin Is Required for ERLAD

To understand whether MAN1B1 relies on any ER‐phagy receptors to degrade class I fusion proteins, we knocked out all those known ER‐phagy receptors in HEK293T cells by CRISPR/Cas9 (Figure S1A, Supporting Information). However, we found that MAN1B1 could still decrease class I fusion protein expression in these KO cell lines (Figure S1B,C, Supporting Information).

**Figure 3:**
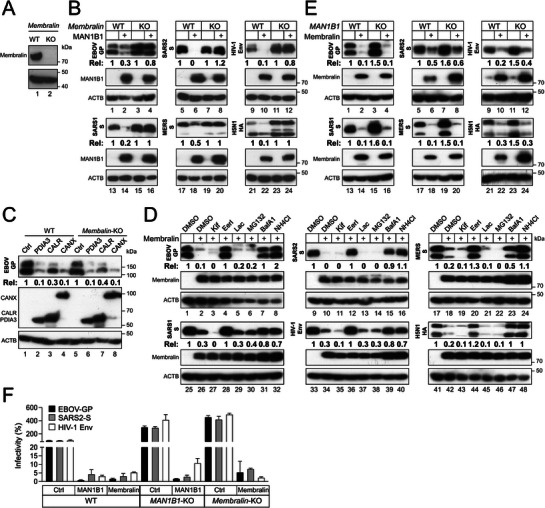
Membralin is required for class I fusion protein degradation. A) Membralin was knocked out in HEK293T cells by CRISPR/Cas9 and confirmed by WB. B) Class I fusion proteins were expressed with MAN1B1 in HEK293T WT and *Membralin*‐KO cells, and their expression was determined by WB. C) EBOV‐GP was expressed with indicated ER chaperones PDIA3, CALR, and CANX in HEK293T WT and *Membralin*‐KO cells, and their expression was determined by WB. D) Class I fusion proteins were expressed with Membralin in HEK293T cells, and treated with 50 µm Kif, 10 µm Eerl, 20 µm Lac, 20 µm MG132, 100 nm BafA1, or 20 mm NH_4_Cl. Protein expression was determined by WB. E) Class I fusion proteins were expressed with Membralin in HEK293T WT and *MAN1B1*‐KO cells, and their expression was determined by WB. F) PVs expressing HIV‐1‐Env, EBOV‐GP, or SARS2‐S were produced from HEK293T WT, *MAN1B1*‐KO, and *Membralin*‐KO cells in the presence or absence of ectopic MAN1B1 or TMEM239 expression. Viral infectivity was determined in TZM‐bI cells for PVs expressing HIV‐1 Env, or in Huh7 cells for PVs expressing EBOV‐GP or SARS2‐S. Results are presented as relative values, with the activity in the presence of a control vector (Ctrl) set as 100. Error bars represent the SEMs calculated from three experiments. The levels of class I fusion protein expression in (B), (C), (D), and (E) were quantified and expressed as Rel values. All experiments were repeated three times, and representative results are shown.

Subsequently, we knocked out another ER transmembrane protein that plays a role in the ERQC, Membralin in HEK293T cells (**Figure** **3**A), which blocked the MAN1B1 activity (Figure 3B, lanes 3, 4, 7, 8, 11, 12, 15, 16, 19, 20, 23, 24). However, ER chaperones PDIA3, calnexin (CANX), and CALR continued to decrease EBOV‐GP expression in this KO cell line (Figure 3C). Thus, MAN1B1 depends on Membralin to inhibit class I fusion protein expression.

Next, we determined whether Membralin can target these viral proteins alone. Indeed, ectopic Membralin strongly decreased their expression (Figure 3D, lanes 1, 2, 9, 10, 17, 18, 25, 26, 33, 34, 41, 42). This decrease was blocked by BafA1, NH_4_Cl, and the p97/VCP inhibitor Eeyarestatin I (EerI) but not by MG132, Lac, and Kif (Figure 3D). To confirm the MAN1B1 independence, we expressed Membralin with class I fusion proteins in HEK293T WT and *MAN1B1*‐KO cells. We found that Membralin still decreased the class I protein expression in this KO cell line (Figure 3E). These results demonstrate that Membralin can inhibit class I fusion protein expression via ERLAD in the absence of MAN1B1.

To demonstrate that the MAN1B1‐Membralin axis has an antiviral activity, FLuc‐PVs expressing EBOV‐GP, SARS2‐S, and HIV‐1‐Env were produced from HEK293T WT, *MAN1B1*‐KO, or *Membralin*‐KO cells in the presence of ectopic MAN1B1 or Membralin expression. The infectivity of all these PVs was increased 3 to 4‐fold when they were produced from *MAN1B1*‐KO or *Membralin*‐KO cells (Figure 3F, compare Ctrl lanes in three cell lines). Conversely, their infectivity was decreased ∼20‐fold by ectopic MAN1B1 or Membralin. We previously reported the potent MAN1B1 antiviral activity against authentic IAV and HIV‐1.^[^ ^12^, ^15^, ^16^ ^]^ Collectively, these results demonstrate that MAN1B1 and Membralin have a broad antiviral activity by targeting class I fusion proteins to ERLAD.

### 2.4 Functional Domain Mapping of the Membralin Cytoplasmic Tail

**Figure 4:**
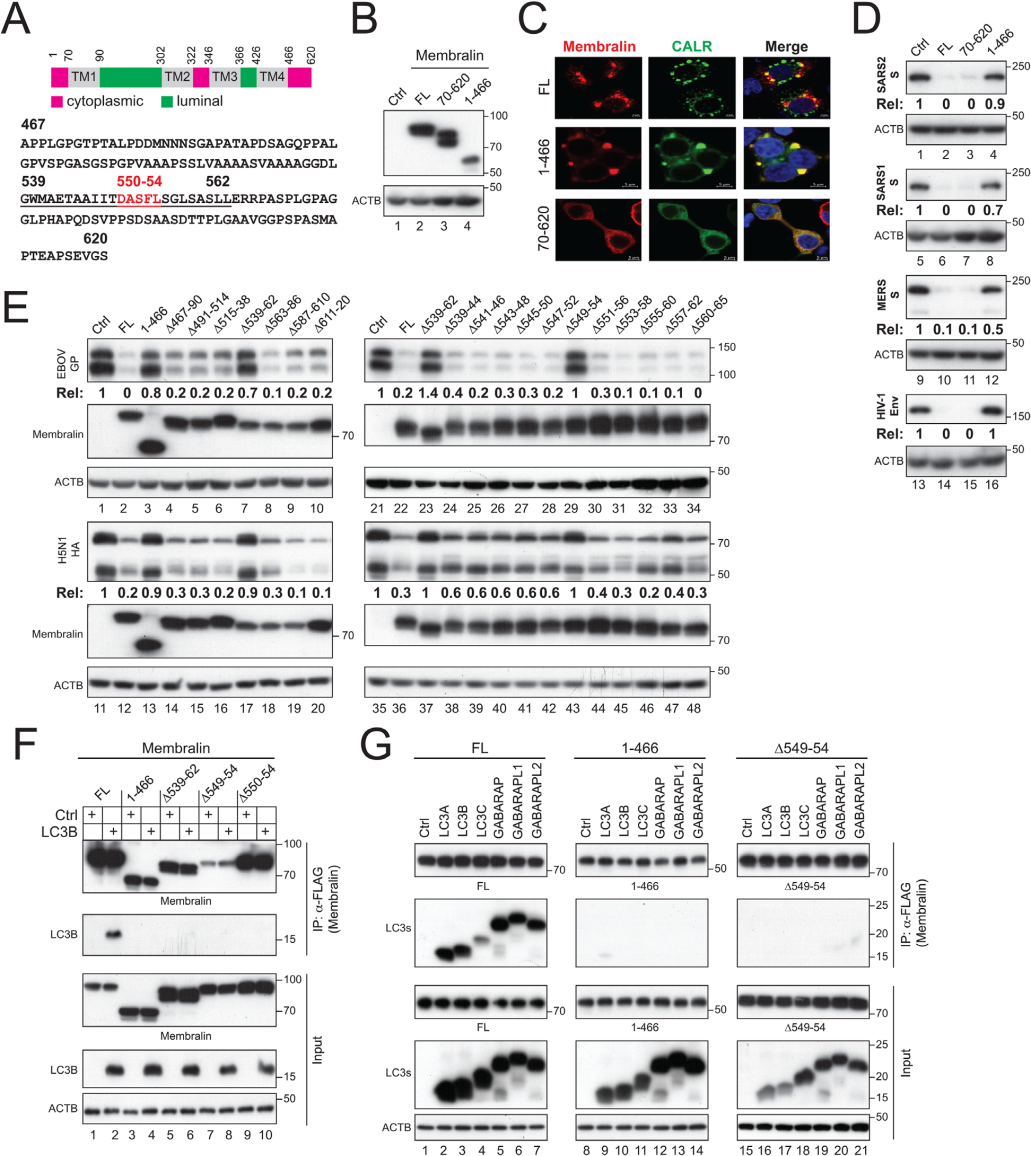
Functional domain mapping of the Membralin cytoplasmic tail. A) A schematic representation of Membralin is shown, featuring four transmembrane (TM) domains (gray), three cytoplasmic domains (pink), and two luminal domains (green), all indicated by their corresponding amino acid (aa) numbers. The sequence of the cytoplasmic tail (aa 467–620) is also displayed, with the region spanning amino acids 539–562 underlined and the ^550^DASFL^554^ motif highlighted in red. B) The expression of indicated Membralin and mutant proteins was determined by WB. FL, the full‐length protein. C) CALR‐GFP was expressed with indicated Membralin proteins with a FLAG‐tag in HeLa cells. Cells were stained with anti‐FLAG followed by Alexa Fluor 647‐conjugated goat anti‐mouse IgG, and their colocalization was determined by confocal microscopy (scale bar 5 µm). D) The indicated Membralin proteins were expressed with class I fusion proteins in HEK293T cells, and their expression was determined by WB. E) The indicated Membralin mutants were expressed with EBOV‐GP and H5N1‐HA, and their expression was analyzed by WB. F) The indicated Membralin mutants with a FLAG‐tag were expressed with LC3B with an HA‐tag in HEK293T cells. Proteins were pulled down by anti‐FLAG and analyzed by WB. G) Membralin and mutants 1–466 and ∆549‐554 with a FLAG‐tag were expressed with LC3/GABARAP family proteins with an HA‐tag in HEK293T cells. Proteins were pulled down by anti‐FLAG and analyzed by WB. The levels of class I fusion protein expression in (D) and (E) were quantified and expressed as Rel values. All experiments were repeated three times, and representative results are shown.

Membralin contains four transmembrane domains, connected by one large and one small luminal loop (**Figure** **4**A, aa 91–301 and 367–425, green) and a relatively short cytoplasmic loop (aa 323–345, pink). To investigate how Membralin mediates ERLAD, we generated two deletion mutants: 70–620 and 1–466, which lack the N‐terminal and C‐terminal cytoplasmic domains, respectively. Western blot analysis showed that both mutants were expressed as the full‐length (FL) protein (Figure 4B). Confocal microscopy further confirmed that all constructs colocalized with the ER marker CALR (Figure 4C), indicating that the deletions did not disrupt Membralin's expression or ER localization. However, when co‐expressed with SARS2‐S, SARS1‐S, MERS‐S, or HIV‐1 Env, the 1–466 mutant failed to reduce their expression levels (Figure 4D, lanes 4, 8, 12, 16).

To screen the critical domain in the cytoplasmic tail (aa 467–620), we created six 24‐aa‐deletion mutants (∆467‐90, ∆491‐514, ∆515‐38, ∆539‐62, ∆563‐86, and ∆587‐610) and one 10‐aa‐deletion mutant (∆611‐20). When these seven mutants were expressed alongside EBOV‐GP and H5N1‐HA, only mutant ∆539‐62 lost the ERLAD activity (Figure 4E, lanes 7, 17). To further narrow down this 24‐aa region, we constructed 11 overlapping 6‐aa‐deletion mutants, including ∆539‐44, ∆541‐46, ∆543‐48, ∆545‐50, ∆547‐52, ∆549‐54, ∆551‐56, ∆553‐58, ∆555‐60, ∆557‐62, and ∆560‐65. When expressed with EBOV‐GP and H5N1‐HA, only mutant ∆549‐54 lost the ERLAD activity (Figure 4E, lanes 29, 43).

The 549–554 region contains 6 residues TDASFL (Figure 4A). We were interested to know whether it contains an LC3‐interacting region (LIR), which is a short peptide sequence that serves as a binding site for LC3/GABARAP proteins crucial for autophagy. To gain further insight into this short region, we created another 5‐aa‐deletion mutant ∆550‐54 by deleting DASFL.

We expressed Membralin FL protein and mutants 1–466, ∆539‐62, ∆549‐54, and ∆550‐54 with a FLAG‐tag alongside LC3B with an HA‐tag and assessed their interactions through immunoprecipitation. The FL protein successfully pulled down LC3B, but all these deletion mutants did not (Figure 4F), indicating ^550^DASFL^554^ is required for LC3B‐binding. To further confirm its critical role, we expressed the FL, 1–466, and ∆549‐54 alongside LC3A, LC3B, LC3C, GABARAP, GABARAPL1, and GABARAPL2 and repeated this experiment. The FL protein bound to all these different LC3 proteins, but 1–466 and ∆549‐54 did not (Figure 4G). Collectively, these results identify ^550^DASFL^554^ as a critical motif for LC3‐binding.

### 2.5 Membralin Recruits VCP and MAN1B1 Viral Different Regions

The Membralin's ERLAD activity is blocked by EerI (Figure 3D), which is an inhibitor for p97/VCP and SEC61.^[^ ^26^ ^]^ The SEC61 complex consists of α, β, and γ subunits that form a transmembrane channel responsible for protein translocation across the ER membrane.^[^ ^27^ ^]^ In our study, the α subunit was silenced using siRNAs, and the β subunit was knocked out by CRISPR/Cas9 in HEK293T cells (Figure S2A,B, Supporting Information). When class I fusion proteins were co‐expressed with MAN1B1 or Membralin, their expression was still suppressed in both the silenced and KO cells (Figure S2A,C, Supporting Information). These findings exclude SEC61 from ERLAD.

**Figure 5:**
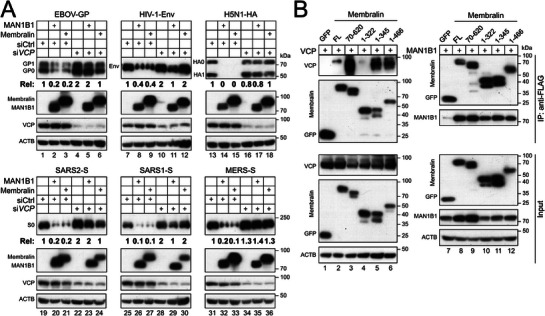
Membralin recruits VCP and MAN1B1 viral different regions. A) Class I fusion proteins were expressed alongside MAN1B1 or Membralin in HEK293T cells in the presence of siRNAs targeting *VCP* or a control (Ctrl). Protein expression levels were assessed by WB. The levels of class I fusion protein expression were quantified and expressed as Rel values. B) VCP‐His and MAN1B1‐HA were expressed with the indicated Membralin deletion mutants or GFP as a control in HEK293T cells. Proteins were pulled down by anti‐FLAG beads. Proteins in the pulldown samples (IP) and cell lysate (Input) were detected by WB. All experiments were repeated three times, and representative results are shown.

Next, we silenced VCP in HEK293T cells by siRNAs and tested the MAN1B1 and Membralin activity. The VCP siRNAs disrupted the MAN1B1 and Membralin activity to class I fusion proteins, but a control siRNA did not (**Figure** **5**A). Thus, VCP is required for the MAN1B1 and Membralin activity.

To understand how VCP interacts with Membralin, we constructed two other Membralin deletion mutants to express its aa 1–322 and 1–345. Together with FL, 1–466, 70–620, and a negative control GFP, they were expressed with VCP in HEK293T cells, and their interactions were determined by immunoprecipitation. The FL, 70–620, 1–345, and 1–466 all pulled down VCP, whereas 1–322 and GFP did not (Figure 5B, lanes 1–6), indicating that VCP binds to the cytoplasmic loop of Membralin (aa 322–346).

We then used the same Membralin constructs to pull down MAN1B1. They all pulled down MAN1B1, except for GFP (Figure 5B, lanes 7–12). These results suggest that MAN1B1 should bind to the large luminal loop of Membralin (aa 90–302).

### 2.6 Membralin Is Not Required for the Degradation of Domestic Misfolded Proteins

Mutations on human genes encoding AAT,^[^ ^28^ ^]^ collagen α‐1 (II) chain (COL2A1),^[^ ^29^ ^]^ Niemann‐Pick type C1 (NPC1),^[^ ^30^ ^]^ and dysferlin (DYSF)^[^ ^31^, ^32^ ^]^ can cause their misfolding in the ER, leading to degradation by ERQC and various genetic diseases.

MAN1B1 targets AAT variant NHK to ERAD,^[^ ^22^, ^33^ ^]^ but targets variant Z (ATZ) to ERLAD.^[^ ^34^ ^]^ NPC1 variant I1061T is targeted to ERAD,^[^ ^35^ ^]^ or ERLAD, via the ER‐phagy receptor RETREG1.^[^ ^36^ ^]^ CANX also targets COL2A1 variant R989C/G1152D to ERLAD via RETREG1.^[^ ^37^ ^]^ The DYSF variant L1341P is targeted to either ERAD or ERLAD.^[^ ^38^ ^]^ Additionally, the autocrine motility factor receptor (AMFR) targets human CD3δ (CD3D) to ERAD,^[^ ^39^ ^]^ and nicastrin (NCSTN) in the γ‐secretase complex is also targeted to ERAD.^[^ ^10^ ^]^ We collectively tested whether the MAN1B‐Membralin axis could target these human proteins.

**Figure 6:**
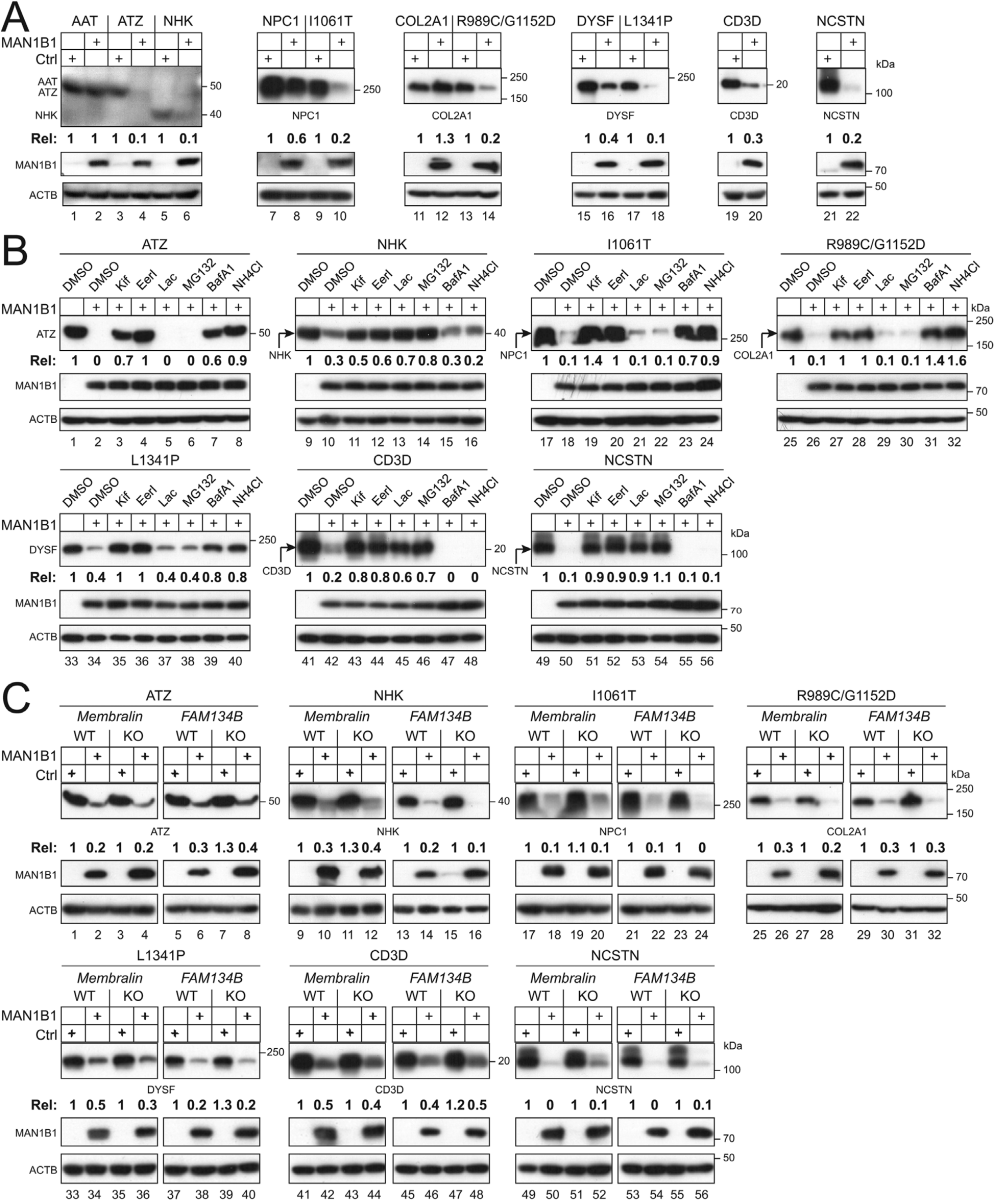
The Membralin‐MAN1B1 axis does not target well‐defined human misfolded or short‐lived proteins. A) AAT and its variants ATZ and NHK, NPC1 and its variant I1061T, collagen alpha‐1(II) chain (CO2A1) and its variant R989C/G1152D, dysferin (DYSF) and its variant L1341P, CD3δ (CD3D), and nicastrin (NCSTN) were expressed with MAN1B1 in HEK293T cells and their expression was determined by WB. B) The indicated misfolded and short‐lived proteins were expressed with MAN1B1 in HEK293T cells. Cells were treated with 50 µm Kif, 10 µm EerI, 20 µm Lac, 20 µm MG132, 100 nm BafA1, or 20 mm NH_4_Cl. Protein expression was determined by WB. C) The indicated misfolded and short‐lived proteins were expressed with MAN1B1 in HEK293T WT, *Membralin*‐KO, and *RETREG1 (RETR1)*‐KO cells. Protein expression was determined by WB. The expression levels of these human proteins were quantified and expressed as Rel values. All experiments were repeated three times, and representative results are shown.

First, we expressed AAT and its variants ATZ and NHK, NPC1 and its variant I1061T, COL2A1 and its variant R989C/G1152D, DYSF and its variant L1341P, CD3δ, and NCSTN with MAN1B1 in HEK293T cells. MAN1B1 effectively decreased the ATZ, NHK, 1061T, R989C/G1152D, L1341P, CD3δ, and NCSTN expression (**Figure** **6**A, lanes 4, 6, 10, 14, 18, 20, 22), but much less effectively the WT AAT, NPC1, COL2A1, and DYSF expression (lanes 2, 8, 12, 16).

Second, we determined how these misfolded variants and the short‐lived CD3δ and NCSTN proteins are degraded. All their decreases by MAN1B1 were blocked by Kif and EerI (Figure 6B, lanes 3, 4, 11, 12, 19, 20, 27, 28, 35, 36, 43, 44, 51, 52). The decrease of NHK, CD3δ, and NCSTN was blocked by Lac and MG132, indicating that they are targeted to ERAD (lanes 13, 14, 45, 46, 53, 54). The decrease of ATZ, I1061T (NPC1), R989C/G1152D (COL2A1), and L1341P (DYSF) was blocked by BafA1 and NH4Cl, indicating that they are targeted to ERLAD (lanes 7, 8, 23, 24, 31, 32, 39, 40).

Third, we determined whether Membralin is required. These proteins were expressed with MAN1B1 in HEK293T WT and *Membralin*‐KO cells. Because RETREG1 (FAM134B) serves as the ER‐phagy receptor for R989C/G1152D (COL2A1) and I1061T (NPC1), the *FAM134B*‐KO cell line was also included. Notably, none of their degradation was blocked in *Membralin*‐KO and *FAM134B*‐KO cells (Figure 6C).

### 2.7 Selection of ER Membrane Proteins for ERLAD

Surprisingly, RETREG1 (FAM134B) is not required for MAN1B1‐mediated degradation of I1061T (NPC1) and R989C/G1152D (COL2A1) (Figure 6C, lanes 23, 24, 31, 32). To understand how ER‐phagy receptors are selected, ATZ, I1061T (NPC1), R989C/G1152D (COL2A1), and L1341P (DYSF) were expressed with MAN1B1 in HEK293T WT cells and six ER‐phagy receptor KO cell lines. MAN1B1 still effectively decreased their expression in these KO cells, indicating that these ER‐phagy receptors are not required for the MAN1B1 activity (Figure S3, Supporting Information).

**Figure 7:**
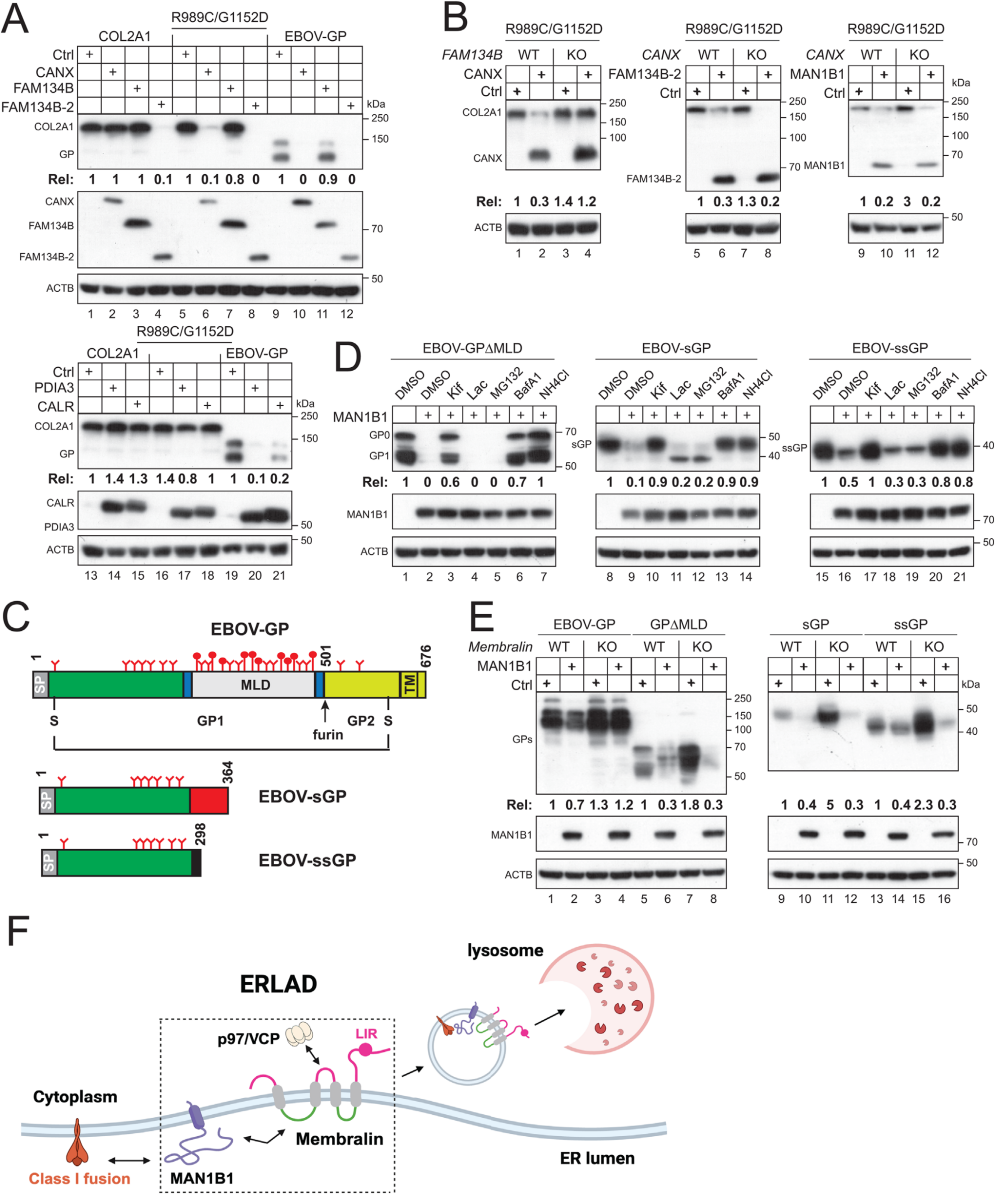
Multiple factors determine the specific degradation. A) COL2A1, its variants R989C/G1152D, and EBOV‐GP were co‐expressed with CANX, PDIA3, CALR, RETREG1, and RETREG1‐2 in HEK293T cells. Protein expression levels were assessed by WB. B) The R989C/G1152D variant was expressed with CANX, RETREG1‐2, or MAN1B1 in HEK293T WT, *RETR1*‐KO, or *CANX*‐KO cells. Protein expression was determined by WB. C) A schematic representation of EBOV glycoproteins is shown, including GP, sGP, and ssGP. The structural glycoprotein is processed into GP_1_ and GP_2_ by furin and linked by a disulfide bond. The letter “Y” indicates *N*‐glycosylation sites, while ⚲ indicates *O*‐glycosylation sites. MLD refers to the mucin‐like domain, and TM indicates the transmembrane domain. D) The indicated EBOV glycoproteins were expressed alongside MAN1B1 in HEK293T cells and treated with the specified inhibitors. Protein expression levels were determined by WB. E) The indicated EBOV glycoproteins were expressed with MAN1B1 in HEK293T WT and *Membralin*‐KO cells, and protein expression was assessed by WB. F) Proposed model of class I fusion protein degradation mediated by MAN1B1 and Membralin. During productive viral infection, heavily glycosylated class I fusion proteins are recognized by MAN1B1, which facilitates their extensive demannosylation within the ER. The resulting low‐mannose glycoproteins are then directed by the Membralin–MAN1B1 complex to a specific ER subdomain. From this site, they are extracted by the AAA‐ATPase p97/VCP and subsequently targeted for ERLAD. The precise mechanisms underlying ER‐phagy in this pathway remain to be elucidated. The expression levels of indicated human proteins and EBOV‐GPs in (A), (B), (D), and (E) were quantified and expressed as Rel values. All experiments were repeated three times, and representative results are shown.

To further explore this mechanism, we investigated the degradation of the COL2A1 variant R989C/G1152D, which is mediated by the ER chaperone CANX via FAM134B. As comparisons, we included EBOV‐GP, that can be targeted to ERLAD by CANX, CALR, and PDIA3. Consistently, CANX, CALR, and PDIA3 decreased EBOV‐GP expression (**Figure** **7**A, lanes 10, 20, 21), as previously reported,^[^ ^14^, ^25^ ^]^ and CANX decreased R989C/G1152D but not WT COL2A1 expression (lanes 2, 6), as reported.^[^ ^37^ ^]^ FAM134B did not decrease any COL2A1, R989C/G1152D, and EBOV‐GP expression (lanes 3, 7, 11), but FAM134B‐2 decreased all these protein expression (lanes 4, 8, 12).

Additionally, CANX did not decrease R989C/G1152 expression in *FAM134B*‐KO cells (Figure 7B, lanes 3, 4), supporting that FAM134B is the ER‐phagy receptor for this degradation.^[^ ^37^ ^]^ FAM134B‐2 and MAN1B1 still decreased R989C/G1152D expression in *CANX*‐KO cells, indicating that their activity is independent of CANX (lanes 5–12). These results demonstrate that the selection of ER membrane protein is determined by both effector (MAN1B1, CANX, FAM134B‐2) and client proteins (host and viral proteins), highlighting the specificity of the Membralin‐MAN1B1 axis in class I fusion protein targeting.

### 2.8 The Membralin‐MAN1B1 Axis Senses Dense Glycans

In addition to the structural GP, EBOV expresses soluble GP (sGP) and small soluble GP (ssGP) via RNA editing,^[^ ^14^, ^25^ ^]^ all sharing the same N‐terminal domain (Figure 7C). EBOV‐GP is heavily glycosylated, particularly in the mucin‐like domain (MLD).^[^ ^40^ ^]^ It has 17 N‐glycosylation sites, 8 of which are in MLD. In addition, MLD contains over 80 O‐glycosylation sites, resulting in higher GP_1_ molecular weight than the precursor GP_0_ (Figure 2D, lanes 1–6; Figure 2E, lanes 1–4). To understand how glycans contribute to GP degradation, we tested the degradation of GP deletion mutant GP∆MLD that does not express MLD, along with sGP and ssGP. MAN1B1 decreased their expression, which was blocked by Kif, BafA1, and NH4Cl, but not Lac and MG132 (Figure 7D). Thus, MAN1B1 could still target these less‐glycosylated GP to lysosomes for degradation. However, when EBOV‐GP, GP∆MLD, sGP, and ssGP were expressed with MAN1B1 in *Membralin*‐KO cells, only the decrease of FL EBOV‐GP was blocked, whereas the others were not (Figure 7E, lanes 3, 4). These results support a hypothesis that dense glycans are required for Membralin‐MAN1B1 to target EBOV‐GP for degradation.

## 3 Discussion

The antiviral activity of MAN1B1 was first identified in human CEM CD4⁺ T cells, where it strongly restricted HIV‐1 infection at the virus entry stage during subsequent rounds of infection.^[^ ^13^, ^17^ ^]^ A key observation was the rapid Env protein turnover, revealed by an S^3^⁵ pulse‐chase assay,^[^ ^15^ ^]^ which led to the discovery of Env as the first viral substrate targeted for MAN1B1‐mediated degradation.^[^ ^16^ ^]^ Later studies revealed that other viral glycoproteins—such as IAV‐HA and EBOV‐GP—are also substrates of MAN1B1.^[^ ^12^, ^14^ ^]^ In this study, we show that MAN1B1 also targets spike proteins from highly pathogenic human coronaviruses, further underscoring its broad antiviral role in degrading viral glycoproteins.

Our study identifies Membralin as a new ER‐resident scaffold that assembles a MAN1B1–VCP degradation complex to initiate a selective branch of ERLAD. This machinery targets viral class I fusion glycoproteins—such as SARS spike, Ebola GP, influenza HA, and HIV‐1 Env—but not typical misfolded host glycoproteins. These findings reveal an unrecognized antiviral quality‐control axis at the ER–lysosome interface and expand the functional repertoire of MAN1B1 beyond canonical ERAD.

We demonstrate that Membralin functions as a molecular scaffold linking luminal and cytosolic degradation events. Its large luminal loop binds MAN1B1, the initiating α‐mannosidase that trims high‐mannose N‐glycans to signal disposal, while its cytoplasmic loop recruits VCP/p97, a segregase that drives membrane remodeling and vesicular delivery. Importantly, Membralin contains a noncanonical LIR (^550^DASFL^554^) that bridges the ER degradation machinery to the autophagy–lysosome system. Deletion of this motif abolishes LC3 binding and ERLAD activity, establishing Membralin as a bona fide ER‐phagy receptor. This organization parallels that of other ER‐phagy receptors (e.g., FAM134B, RTN3L, and TEX264) yet is distinguished by its substrate specificity and requirement for MAN1B1.

Our data reveal that the MAN1B1–Membralin axis functions independently of polyubiquitination, in contrast to PDIA3‐mediated degradation of EBOV‐GP. Neither MAN1B1 nor Membralin induced ubiquitin conjugation of class I fusion proteins, and proteasome inhibitors failed to block degradation. Instead, lysosomal and autophagy inhibitors fully rescued substrate expression. This suggests that MAN1B1–Membralin defines a ubiquitin‐independent ERLAD route, directly coupling luminal glycan trimming to vesicular sequestration and lysosomal degradation. The requirement of VCP further supports a model in which cytosolic extraction or membrane budding is coordinated by the Membralin–VCP complex.

Unlike conventional ERQC substrates such as AAT, NPC1, COL2A1, and DYSF variants, viral fusion proteins were uniquely dependent on Membralin for degradation. These viral glycoproteins share a heavily glycosylated trimeric architecture that masks immunogenic epitopes and stabilizes prefusion conformations. We show that dense N‐glycan clustering, rather than overall folding defects, could be the critical signal sensed by Membralin–MAN1B1. Deletion of the mucin‐like domain in EBOV‐GP or expression of less glycosylated sGP and ssGP abrogated the requirement for Membralin, implying that Membralin selectively recognizes substrates with high glycan density. This specialization may have evolved as a host defense mechanism to counteract viral exploitation of the ER folding machinery.

We further show that none of the established ER‐phagy receptors (FAM134A/B/C, TEX264, RTN3L, ATL3, CCPG1, or SEC62) are required for MAN1B1‐ or Membralin‐mediated degradation, underscoring the existence of an independent ERLAD branch. Membralin's structural topology—four transmembrane segments with dual luminal and cytoplasmic loops—enables simultaneous engagement of luminal MAN1B1 and cytosolic LC3/VCP. This dual topology likely endows Membralin with higher substrate selectivity and may explain its unique antiviral preference. By contrast, canonical ER‐phagy receptors primarily mediate turnover of general ER portions or misfolded host proteins via reticulon‐homology domains.

The Membralin–MAN1B1 axis provides a new paradigm in ER quality control—one that discriminates foreign glycoproteins from self‐misfolded proteins. Because many enveloped viruses rely on the host ER for glycoprotein maturation, this pathway likely represents an intrinsic cellular defense that limits viral assembly by diverting spike or envelope proteins to lysosomes. Indeed, knockout of either MAN1B1 or Membralin markedly enhanced pseudoviral infectivity, whereas their overexpression potently suppressed infection. Given that Membralin is essential for neuronal survival and γ‐secretase stability, its dual roles in neuroprotection and antiviral defense may converge on ER homeostasis and stress adaptation.

In summary, our findings define Membralin as an ER‐resident scaffold that coordinates MAN1B1 and VCP to form a dedicated ERLAD complex for selective clearance of viral fusion glycoproteins (Figure 7F). This ubiquitin‐independent pathway extends the landscape of ER quality control and reveals a mechanistic link between glycan density sensing and autophagic degradation. Future studies should elucidate how Membralin distinguishes foreign glycoproteins at the structural level, whether its activity is regulated by innate immune signaling, and how pathogens may evolve to evade this antiviral ERLAD pathway.

## 4 Experimental Section

### Antibodies and Inhibitors

Commercial reagents include: Kifunensine, Eeyarestatin I, MG132, Lactacystin (Lac), Concanamycin A (ConA), 3‐Methyladenine (3‐MA), and ammonium chloride (NH_4_Cl) (Sigma–Aldrich, K1140, E1286, C2211, L6785, C9705, M9281, A9434); Bafilomycin A1 (BafA1, Santa Cruz Biotechnology, sc‐201550); mouse monoclonal anti‐Myc (CST, 2276S); rabbit polyclonal anti‐SQSTM1, mouse monoclonal anti‐FLAG and anti‐HA (Sigma‐Aldrich, P0067, F3165, H3663); mouse monoclonal anti‐ACTB/β‐actin (CST, 3700S); mouse monoclonal anti‐ERManI/MAN1B1 (Novus, NBP2‐13167); rabbit polyclonal anti‐RETREG1‐2 and anti‐Membralin (Gene Tex, GTX46621, GTX118618); rabbit polyclonal anti‐ATL3, anti‐RETREG1, anti‐CCPG1, anti‐TEX264, and anti‐RETREG2 (Proteintech, 16921‐1‐AP, 21537‐AP, 13861‐1‐AP, 25858‐1‐AP, 24650‐1‐AP); rabbit monoclonal anti‐SEC62 and rabbit polyclonal anti‐EDEM2 (Abcam, ab137022, ab181218); rabbit polyclonal anti‐RTN3 (ThermoFisher Scientific, PA5‐78316); mouse monoclonal anti‐SERPINA1/AAT (Sigma‐Aldrich, SAB4200198); rabbit polyclonal anti‐Sec61A and Sec61B (Abcam, ab183046, ab15576); rabbit polyclonal anti‐VCP (Huabio, ER30603); horseradish peroxidase (HRP)‐conjugated goat anti‐mouse IgG and anti‐rabbit IgG (Jackson ImmunoResearch Laboratories, 115‐035‐003, 111‐035‐003).

### Cells

Human embryonic kidney (HEK) 293 cell line transformed with SV40 large T antigen (HEK293T) and human cervical carcinoma cell line HeLa were purchased from American Type Culture Collection (ATCC, CRL‐3216, CRM‐CCL‐2); human hepatoma cell line Huh7 was purchased from BioBW, China (BioBW, bio‐73061); TZM‐bI cells were from NIH HIV Reagent Program. The exact purchase dates of these cell lines could not be verified, but this does not affect the conclusions. All cell lines were confirmed to be free of contamination.

All these cells were maintained in Dulbecco's modified Eagle medium (DMEM; Thermo Fisher Scientific, 11 965 092) supplemented with 10% fetal bovine serum (FBS) and 1% penicillin‐streptomycin (pen‐strep; Thermo Fisher Scientific, 10 378 016) and cultivated at 37 °C in the humidified atmosphere in a 5% CO2 incubator.

### CRISPR‐Cas9 Knockout

DNA oligos encoding small guide RNAs (sgRNAs) targeting MAN1B1, EDEM2, Membralin (TMEM259), RETREG2, RTN3L, ATL3, SEC62, CCPG1, TEX264, Sec61B, and RETREG2 are shown in Table S1 (Supporting Information), which were cloned into the pSpCas9(BB)‐2A‐GFP (PX458) vector via BbsI digestion. HEK293T cells were transfected with these vectors, and after 48 h, single clones of GFP‐positive cells were isolated by fluorescence‐activated cell sorting. Knockout clones were identified by Western blotting and further validated by DNA sequencing. The HEK293T RETREG3 (RETR3)‐knockout cell line was just reported.^[^ ^41^ ^]^

### siRNA Knockdown

SEC61A and VCP were knocked down by siRNAs using RNA oligo pairs 5′‐CACUGAAAUGUCUACGUUUTT‐3′/5′‐AAACGUAGACAUUUCAGUGTT‐3′, or 5′‐GCAUGUGGGUGCUGACUUATT‐3′/5′‐UAAGUCAGCACCCACAUGCTT‐3′. HEK293T cells were transfected with these siRNAs, and after 24 h, their expression was determined by WB.

### Plasmids

The pcDNA3.1‐hERManI‐HA‐FLAG, pCMV6‐hEDEM1‐Myc‐FLAG, pcDNA3.1‐hEDEM2‐HA‐FLAG, and pCMV6‐hEDEM3‐Myc‐FLAG vectors and ERManI catalytically inactive mutant expression vector were reported previously.^[^ ^12^ ^]^ pcDNA3.1 vectors expressing ERManI/MAN1B1, EDEM1, EDEM2, and EDEM3 with a single C‐terminal HA‐tag were re‐constructed in this study by homologous recombination after NheI/XhoI digestion. pNL‐Luc‐ΔEnv, pcDNA3.1‐FLAG‐EBOV‐GP, pcDNA3.1‐FLAG‐EBOV‐GP∆MLD, pcDNA3.1‐HiBiT‐sGP, pcDNA3.1‐HiBiT‐ssGP, HIV‐1 Env, and H5N1 HA expression vectors were reported previously.^[^ ^14^, ^25^ ^]^ pCAGGS‐PDIA3‐Myc, pCMV‐CALR‐Myc, pCMV6‐CANX‐Myc‐FLAG, HSPA5/BiP promoter‐luciferase reporter vector pLightSwitch‐BiP, XBP1‐activation reporter vector pXBP1u‐FLuc, ATF4‐expression reporter vector pATF4‐UTR‐Fluc, and ATF6‐activation reporter vector p5xATF6‐GL3 were reported previously.^[^ ^14^, ^25^ ^]^ pCMV‐HA‐Ub_WT_ and pCMV‐HA‐Ub_KO_ were reported previously.^[^ ^42^ ^]^ pSpCas9 (BB)‐2A‐GFP (PX458) was obtained from Feng Zhang through Addgene (48 138). pCMV6‐Membralin‐Myc‐FLAG, pCMV6‐RETREG1‐2‐Myc‐Flag, pCMV6‐LC3A‐HA, pCMV6‐LC3B‐HA, pCMV6‐LC3C‐HA, pCMV6‐GABARAP‐HA, pCMV6‐GABARAPL1‐HA, and pCMV6‐GABARAPL2‐HA were ordered from Comatebio (CoME). Membralin deletion mutants were created by PCR and ASiSI/MluI digestion followed by homologous recombination. pCDNA3.1‐RETREG1‐FLAG was ordered from GenScript. pCDNA3.1‐NPC1‐3xFLAG was reported previously.^[^ ^43^ ^]^ pCDNA3.1‐NHK was provided by Richard Sifers. pCMV6‐SERPINA1‐Myc‐FLAG, pCMV6‐COL2A1‐Myc‐FLAG, pCMV6‐DYSF‐Myc‐FLAG, pCMV6‐CD3D‐Myc‐FLAG, pCMV6‐NCSTN‐Myc‐FLAG, and pCMV6‐VCP‐Myc‐FLAG were ordered from Origene (Cat# RC202082, RC218041, RC219485, RC210010, RC212646, RC211130). SERPINA1/AAT, NPC1, COL2A1, and DYSF mutants and pCMV6‐VCP‐His were created by PCR and ASiSI/MluI digestion followed by homologous recombination. All vectors constructed were confirmed by Sanger DNA sequencing. Detailed experimental procedures and primer sequences for the construction of these vectors are available upon request. Plasmids were prepared using Maxiprep kits (TIANGEN Biotech, DP117).

To express S proteins from SARS2, SARS1, and MERS, their cDNAs with a N‐terminal FLAG tag were commercially synthesized and cloned into pCAGGS, resulting in pCAGGS‐FLAG‐SARS2‐S, pCAGGGS‐FLAG‐SARS1‐S, and pCAGGS‐FLAG‐MERS‐S (Comate Bioscience, China). A SARS2‐S and SARS1‐S expression vector with a N‐terminal FLAG‐tag and C‐terminal 19 amino acids deletion, pCAGGS‐FLAG‐SRAS2‐S‐D19 and pCAGGS‐FLAG‐SARS1‐S‐D19, were re‐created by EcoRI/XhoI digestion. A SARS1‐S expression vector with a C‐terminal FLAG, pCAGGS‐SARS1‐S‐FLAG, was re‐created by EcoRI/BspEI digestion. These two SARS2‐S and SARS1‐S with this deletion were used to produce HIV‐1 pseudovirions for infectivity assay. To express S protein‐EGFP fusion proteins, these cDNAs were cloned into pEGFP‐HA‐N1 vector expressing an HA‐tag in front of EGFP by XhoI/BspEI digestion, resulting in pEGFP‐SARS2‐S‐HA, pEGFP‐SARS1‐S‐HA, pEFGP‐MERS‐S‐HA, which express SARS2‐S‐HA‐EGFP, SARS1‐S‐HA‐EGFP, and MERS‐S‐HA‐EGFP fusion proteins. To express MAN1B1‐mCherry fusion, MAN1B1 was subcloned from pcDNA3.1‐hMAN1B1‐HA into pCAGGS‐mCherry by EcoRI digestion. pEGFP‐N1 vectors expressing CALR and TGLON2 were constructed by PCR and XhoI/BspEI digestion.

### Transfection

HEK293T cells were cultured in 6‐well plates and transfected with polyethyleneimine (PEI; Polysciences, 23966‐2). HeLa cells were transfected using Lipofectamine 3000 according to the manufacturer's protocol (Thermo Fisher Scientific, L3000015). The total indicated plasmids were diluted into 200 µL serum‐free Opti‐MEM (Thermo Fisher Scientific, 31 985 062) and mixed with transfection reagents. After 20 min of incubation at room temperature, these transfection complexes were added directly into the supernatant of each well. Media were replaced after 6 h, and cell lysate was collected at 48 h unless otherwise noted. Viruses released into the supernatants and proteins expressed in cells were analyzed (see below).

### Analysis of UPR

HEK293T cells were cultured in 24‐well plates and transfected with the indicated plasmid and reporter vectors as reported previously.^[^ ^12^, ^14^ ^]^ HSPA5/BiP activation was measured by expressing BiP‐RLuc reporter construct and measuring the intracellular Renilla luciferase activities. XBP1, ATF4, and ATF6 activation were measured by expressing XBP1‐Fluc, pATF4‐UTR‐Fluc, or p5×ATF6‐GL3 reporter construct and measuring the intracellular Firefly luciferase activities. Luciferase activities were analyzed by Dual‐Luciferase Reporter Assay System (Promega, E1910).

### FLuc‐pseudovirion (PV) Infection

SARS2‐S, SARS1‐S, and MERS‐S pseudotyped HIV‐1 virions were produced from HEK293T WT and *MAN1B1*‐KO cells as reported previously.^[^ ^43^ ^]^ These cells were transfected with pNL‐Luc‐ΔEnv and an S protein expression vector in the presence or absence of a MAN1B1 expression. After 48 h, viruses were collected from the culture supernatants and clarified by low‐speed centrifugation. After being normalized by p24^Gag^ ELISA,^[^ ^44^ ^]^ an equal number of viruses were used to infect Huh7 cells. After 48 h of infection, cells were lysed, and viral infectivity was determined by measuring the intracellular luciferase activity using a firefly luciferase assay kit (US Everbright Inc, Cat# F6024).

### Western Blotting (WB)

Transfected cells were lysed in RIPA buffer (25 mm Tris, pH7.4, 150 mm NaCl, 0.5% sodium deoxycholate, 0.1% SDS, 1%Nonidet P‐40 [Sigma–Aldrich, R0278]) at 4 °C. After centrifugation at 12 000 × g for 10 min at 4 °C, cytosolic fractions were collected and boiled with SDS‐polyacrylamide gel electrophoresis (SDS‐PAGE) loading buffer (Solarbio Life Sciences, P1015). Proteins were separated by SDS‐PAGE and transferred onto PVDF membranes. These membranes were blocked with 5% nonfat milk powder in TBST (Tris‐buffered saline [20 mm Tris, pH 7.4,150 mm NaCl] containing 0.1% Tween 20; Solarbio Life Sciences, T8220) for 1 h at room temperature and probed by primary antibodies followed by HRP‐conjugated secondary antibodies. The blotted proteins were detected by SuperSignal substrate (Thermo Fisher Scientific, 34 580) using X‐ray films (FUJI) as reported.^[^ ^45^ ^]^ Films were scanned, and protein bands were quantified by ImageJ. Adobe Photoshop and Adobe Illustrator were used to generate the figures.

### Immunoprecipitation

After transfection of HEK293T cells cultured in a 6‐cm dish, cells were lysed in 0.8 mL RIPA lysis buffer for 30 min on ice, as did previously.^[^ ^46^ ^]^ After the removal of nuclei via low‐speed centrifugation and collecting 100 µL as input, the remaining 700 µL lysate was incubated with anti‐FLAG M2 Magnetic beads (Sigma‐Aldrich, M8823) and rotated at 4 °C overnight. After being washed 3 times with 1 mL pre‐cooled RIPA lysis buffer, proteins were removed from beads after boiling in 40 µL RIPA lysis buffer plus 15 µL sample loading buffer (5×) and analyzed by WB.

### In Vivo Polyubiquitination Assay

HEK293T cells were seeded in 6‐cm dishes and transfected with vectors expressing SARS2‐S and MAN1B1, or EBOV‐GP and PDIA3, in the presence of ubiquitin expression vector as previously.^[^ ^42^ ^]^ After 48 h, cells in each dish were lysed in 600 µL RIPA buffer at 4 °C for 30 min. After the removal of nuclei by low‐speed centrifugation, 100 µL was collected as input, and the remaining 500 µL was incubated with anti‐FLAG beads (Sigma–Aldrich, M8823) at 4 °C overnight. Beads were washed three times with phosphate‐buffered saline (PBS), then boiled in SDS‐PAGE loading buffer and analyzed by WB.

### Confocal Microscopy

HeLa cells were seeded on a glass‐bottom cell culture dish (NEST Biotechnology, 801 001) and transfected with various vectors using Lipofectamine 3000, as previously.^[^ ^47^ ^]^ After 30 h, cells were fixed with 4% paraformaldehyde for 10 min, permeabilized with 0.1% Triton X‐100 (Solarbio Life Sciences, T8200) for 10 min at room temperature, and then blocked with 5% bovine serum albumin (BSA; APPLYGEN, P1622) solution overnight at 4 °C. Nuclei were stained with 4′,6‐diamidino‐2‐phenylindole (DAPI) for 3–5 min and washed with PBS, and cells were observed and imaged under a confocal microscope (LSM880, Zeiss, White Plains, New York). Quantitative colocalization measurements were performed using ImageJ software. Pearson's correlation coefficient (PCC) was calculated to describe the colocalization correlation of the intensity distributions between two channels.

### Statistical Analysis

All experiments were performed independently at least three times, with representative experiment being shown. GraphPad Prism (Graph Pad Software Inc., San Diego, CA, USA) was used for the data analysis. Data were presented as means ± standard error of measurements (SEMs) and represented by error bars. The significance of differences between samples was assessed by an unpaired two‐tailed Student's t test. A p value < 0.05 (p < 0.05) was statistically significant (*p < 0.05, **p < 0.01, ***p < 0.001), and p > 0.05 was not significant (ns).

## Conflict of Interest

The authors declare no conflict of interest.

## Author Contributions

J.Z. and X.L. contributed equally to this work. J.Z. comleted Figures 2A, 2C, 2G, 3‐7, and supplemental figures S1‐S3 with support from S.L., T. W., and I.A. X,L. completed Figures 1, 2B, and 2D‐2F. J.Z. and Y.H.Z. designed experiments. Y.H.Z. wrote the manuscript with input from all authors.

## Supporting information



Supporting Information

## Data Availability

The data that support the findings of this study are available from the corresponding author upon reasonable request.
