# Supplementary material for: Membralin Assembles a MAN1B1–VCP Complex to Target Foreign Glycoproteins from the Endoplasmic Reticulum to Lysosomes for Degradation
Source: Adv Sci (Weinh). 2025 Dec 1;13(9):e19256. doi: 10.1002/advs.202519256 (PMC12904022; doi:10.1002/advs.202519256)

Table S1. Oligos were used to establish KO cells in this study.

Primer	sense（5′-3′）	antisense（5′-3′）
TMEM259-gRNA1	caccgGGTCGCGCACGTTGATGAG	aaacCTCATCAACGTGCGCGACCc
TMEM259-gRNA2	caccgGAACTCGAAGAGACGGCGGA	aaacTCCGCCGTCTCTTCGAGTTCc
RETREG1-gRNA1	caccgGCTTCCAGCTCAGCAGCTCGT	aaacACGAGCTGCTGAGCTGGAAGCc
RETREG1-gRNA2	caccgGCAATACAGTGGCTGAGCCT	aaacAGGCTCAGCCACTGTATTGCc
RTN3-gRNA1	caccgGAGGCTGGCTCCCTAACACA	aaacTGTGTTAGGGAGCCAGCCTCc
RTN3-gRNA2	caccgGGAGATGGAATGGGACTGAG	aaacCTCAGTCCCATTCCATCTCCc
ATL3-gRNA1	caccgGCCACCACCACATCAAGATCT	aaacAGATCTTGATGTGGTGGTGGCc
ATL3-gRNA2	caccgGTGGTCCTGCAAGAGGATGC	aaacGCATCCTCTTGCAGGACCACc
SEC62-gRNA1	caccgGAAGCTTTATTTACAACCA	aaacTGGTTGTAAATAAAGCTTCc
SEC62-gRNA2	caccgGAACCCGGTGACCCATCATAT	aaacATATGATGGGTCACCGGGTTCc
CCPG1-gRNA1	caccgAGAAACTGCTTATCCAGCTT	aaacAAGCTGGATAAGCAGTTTCTc
CCPG1-gRNA2	caccgGTCTAACTTAGGTGGCTCAA	aaacTTGAGCCACCTAAGTTAGACc
TEX264-gRNA1	caccgGTGATAAGTGCCGATGTGCCG	aaacCGGCACATCGGCACTTATCACc
TEX264-gRNA2	caccgGTGGCCTTTGCCGGGTACTCA	aaacTGAGTACCCGGCAAAGGCCACc
MAN1B1-gRNA1	caccgCAAATCCACCCGTCTTACC	aaacGGTAAGACGGGTGGATTTGc
MAN1B1-gRNA2	caccgAAATCTCAGGTAAGTTCTC	aaacGAGAACTTACCTGAGATTTc
EDEM2-gRNA1	caccgTGCTCATCTGCTCTCCAAGA	aaacTCTTGGAGAGCAGATGAGCAc
EDEM2-gRNA2	caccgCCTGAGAATGGCTGAGGAGG	aaacCCTCCTCAGCCATTCTCAGGc
SEC61B-gRNA1	caccgACCCCCAGTGGCACTAACGT	aaacACGTTAGTGCCACTGGGGGTc
SEC61B-gRNA2	caccgGCGCTCTCCCAGCAAAGCAG	aaacCTGCTTTGCTGGGAGAGCGCc
RETREG2-gRNA1	caccgGTCACGGCGGCCGCGCTCAA	aaacTTGAGCGCGGCCGCCGTGACc
RETREG2-gRNA1	caccgGGCGGTGGCGGGGGTAACAC	aaacGTGTTACCCCCGCCACCGCCc

Fig. S1. MAN1B1 does not engage with known ER-phagy receptors.
(A) Indicated ER-phagy receptor genes were knocked out in HEK293T cells by CRISPR/Cas9, and their expression was confirmed by WB. **(B)** Class I fusion proteins were expressed with MAN1B1 in HKE293T WT and indicated ER-phagy receptor KO cell lines, and their expression was determined by WB. **(C)** Class I fusion and VSV-G proteins were expressed with MAN1B1 in HKE293T WT and *RETR2*-KO and *RETR3*-KO cells, and their expression was determined by WB.

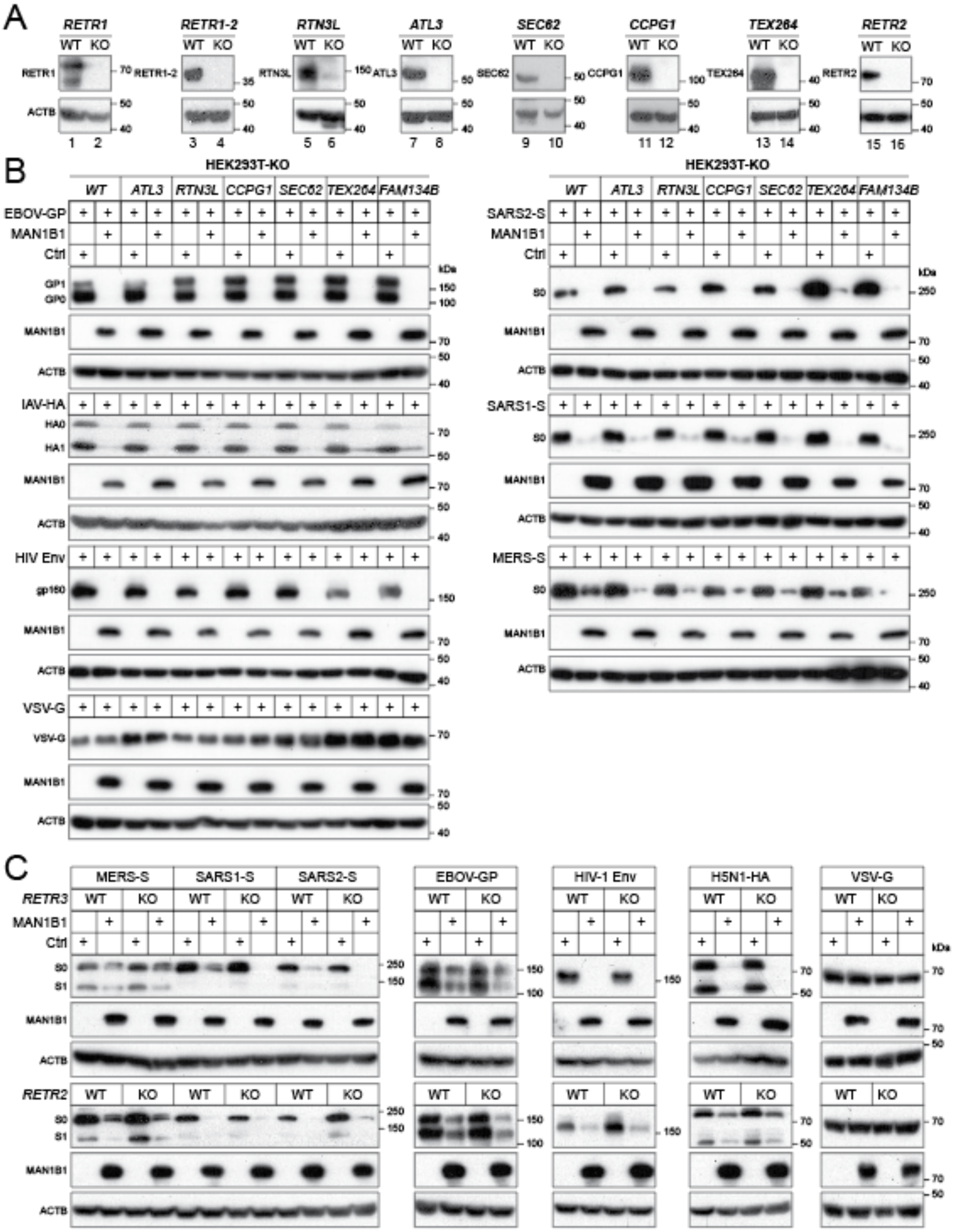

Fig. S2. MAN1B1 and Membralin act independently of the SEC61 translocon.
(A) Class I fusion proteins were expressed with MAN1B1 or TMEM259 in HEK293T cells in the presence of SEC61A siRNAs or a control siRNA, and their expression was determined by WB. **(B)** *SEC61B* was knocked out in HEK293T cells, and two KO clones (B3, B6) were confirmed by WB. **(C)** Class I fusion proteins were expressed with MAN1B1 or Membralin in HEK293T WT or *SEC61B*-KO (B3) cells, and their expression was determined by WB.

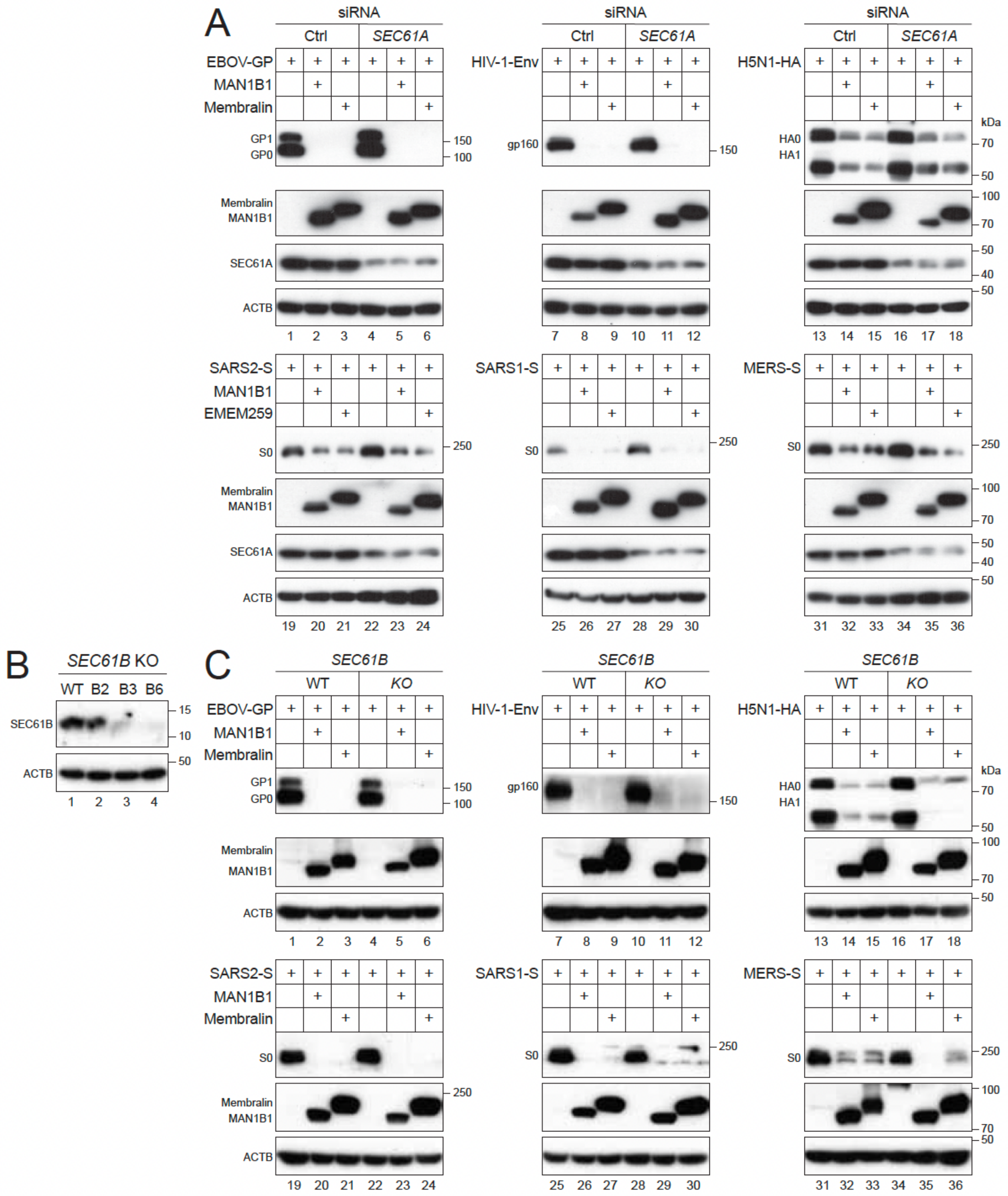

Fig. S3. MAN1B1 does not engage with well-known ER-phagy receptors to degrade misfolded proteins and aggregates.
Indicated misfolded proteins were expressed with MAN1B1 in HEK293T WT and indicated KO cells. Protein expression was determined by WB.

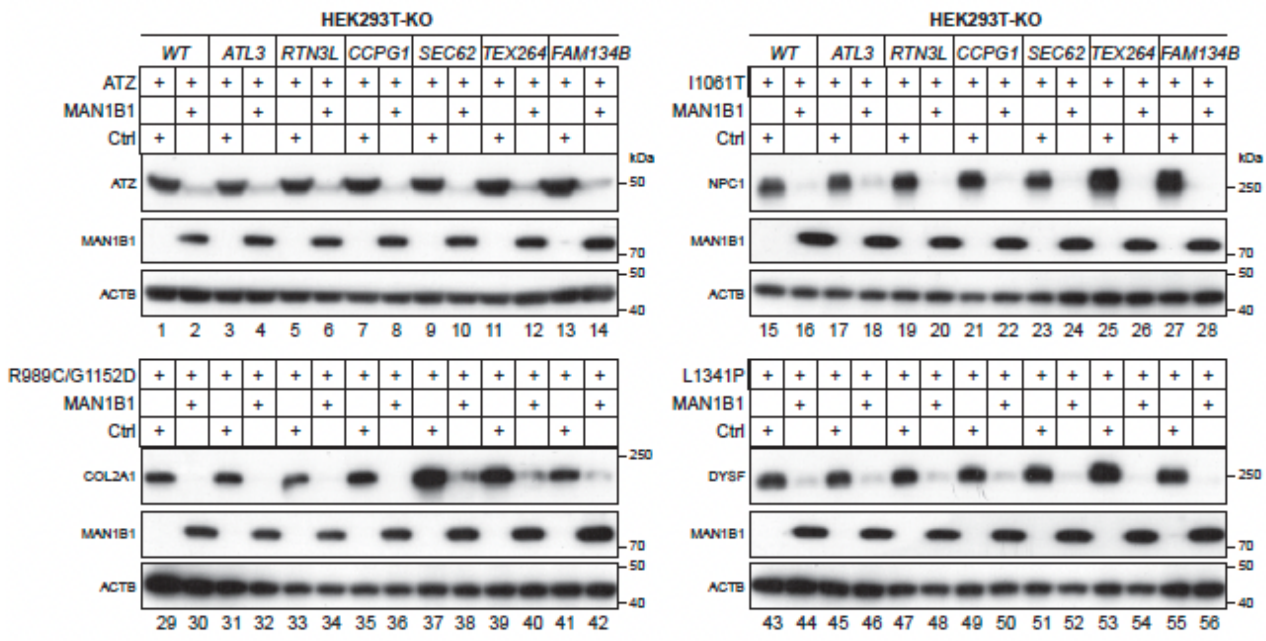

Supplement: Supplementary file 1 — Supporting Information [file ADVS-13-e19256-s001.pdf]
